# 15-hydroxyprostaglandin dehydrogenase (15-PGDH) prevents lipopolysaccharide (LPS)-induced acute liver injury

**DOI:** 10.1371/journal.pone.0176106

**Published:** 2017-04-19

**Authors:** Lu Yao, Weina Chen, Kyoungsub Song, Chang Han, Chandrashekhar R. Gandhi, Kyu Lim, Tong Wu

**Affiliations:** 1 Department of Pathology and Laboratory Medicine, Tulane University School of Medicine, New Orleans, LA, United States of America; 2 Department of Pediatrics, Cincinnati Children’s Hospital Medical Center and Department of Surgery, University of Cincinnati, Cincinnati, United States of America; 3 Department of Biochemistry, College of Medicine, Cancer Research Institute and Infection Signaling Network Research Center, Chungnam National University, Daejeon, Korea; Faculty of Medicine & Health Science, UNITED ARAB EMIRATES

## Abstract

The NAD+-dependent 15-hydroxyprostaglandin dehydrogenase (15-PGDH) catalyzes the oxidation of the 15(S)-hydroxyl group of prostaglandin E_2_ (PGE_2_), converting the pro-inflammatory PGE_2_ to the anti-inflammatory 15-keto-PGE_2_ (an endogenous ligand for peroxisome proliferator-activated receptor-gamma [PPAR-γ]). To evaluate the significance of 15-PGDH/15-keto-PGE_2_ cascade in liver inflammation and tissue injury, we generated transgenic mice with targeted expression of 15-PGDH in the liver (15-PGDH Tg) and the animals were subjected to lipopolysaccharide (LPS)/Galactosamine (GalN)-induced acute liver inflammation and injury. Compared to the wild type mice, the 15-PGDH Tg mice showed lower levels of alanine aminotransferase (ALT) and aspartate aminotransferase (AST), less liver tissue damage, less hepatic apoptosis/necrosis, less macrophage activation, and lower inflammatory cytokine production. In cultured Kupffer cells, treatment with 15-keto-PGE_2_ or the conditioned medium (CM) from 15-PGDH Tg hepatocyes inhibited LPS-induced cytokine production, *in vitro*. Both 15-keto-PGE_2_ and the CM from15-PGDH Tg hepatocyes also up-regulated the expression of PPAR-γ downstream genes in Kupffer cells. In cultured hepatocytes, 15-keto-PGE_2_ treatment or 15-PGDH overexpression did not influence TNF-α-induced hepatocyte apoptosis. These findings suggest that 15-PGDH protects against LPS/GalN-induced liver injury and the effect is mediated via 15-keto-PGE_2_, which activates PPAR-γ in Kupffer cells and thus inhibits their ability to produce inflammatory cytokines. Accordingly, we observed that the PPAR-γ antagonist, GW9662, reversed the effect of 15-keto-PGE_2_ in Kupffer cell *in vitro* and restored the susceptibility of 15-PGDH Tg mice to LPS/GalN-induced acute liver injury *in vivo*. Collectively, our findings suggest that 15-PGDH-derived 15-keto-PGE_2_ from hepatocytes is able to activate PPAR-γ and inhibit inflammatory cytokine production in Kupffer cells and that this paracrine mechanism negatively regulates LPS-induced necro-inflammatory response in the liver. Therefore, induction of 15-PGDH expression or utilization of 15-keto-PGE_2_ analogue may have therapeutic benefits for the treatment of endotoxin-associated liver inflammation/injury.

## Introduction

Endotoxin (Lipopolysaccharides, LPS)-associated liver injury is a significant cause of morbidity and mortality in patients with sepsis and other systematic and hepatic disorders [1,2,3]. Clinical studies have shown that the incidence of endotoxemia associated with acute or chronic hepatitis, fibrosis/cirrhosis and hepatocellular carcinoma reaches as high as 75%-95% [1]. LPS is known to activate liver macrophages (termed Kupffer cells) which initiates an inflammatory response in the liver, contributing to the development of hepatitis and/or liver failure.

Peroxisome proliferator-activated receptor γ (PPAR-γ) is a member of the nuclear hormone receptor superfamily. Following binding to its ligand, PPAR-γ forms a heterodimer with retinoid X receptors (RXRs) to function as a transcription regulator [4,5]. In the liver, PPAR-γ in Kupffer cells is known to inhibit inflammatory cytokine production [4]. Accordingly, mice with disruption of PPAR-γ in Kupffer cells display exacerbated inflammation in response to LPS and show more prominent hepatic tissue damage [6]. Thus, PPAR-γ activation may represent a potential therapeutic strategy to prevent LPS-induced liver inflammation and tissue damage. Indeed, synthetic PPAR-γ ligands (e.g., rosiglitazone) have been shown to reduce LPS-induced cytokine production and liver tissue injury in mice [6].

The NAD+-dependent 15-hydroxyprostaglandin dehydrogenase (15-PGDH) is known to catalyze the oxidation of the 15(S)-hydroxyl group of prostaglandin E_2_ (PGE_2_), converting PGE_2_, a pro-inflammatory and pro-proliferative lipid mediator, to its oxidized product, 15-keto-PGE_2_ [7,8]. 15-keto-PGE_2_ has been long viewed as a biological inactive metabolite of PGE_2_; however, recent studies show that 15-keto-PGE_2_ actually mediates biological function as an endogenous ligand for PPAR-γ [8,9,10,11]. In a mouse model of CFTR-deficiency, regulation of PPAR-γ by 15-PGDH-derived 15-keto-PGE_2_ is reported to be implicated in the pathogenesis of cystic fibrosis [9]. In mouse fibroblasts, 15-PGDH-derved 15-keto-PGE_2_ has been shown to activate PPAR-γ and promote adipocyte maturation [10,11]. In hepatocellular cancer cells, 15-PGDH-derived 15-keto-PGE_2_ has been found to activate PPAR-γ and regulate its downstream genes [8].

In the context of liver, Zhang et al [12] recently described that inhibition of 15-PGDH activity could accelerate liver regeneration after partial hepatectomy; their findings raise a possibility that 15-PGDH inhibition might represent a potential therapeutic strategy to enhance tissue regeneration. However, it remains unknown whether 15-PGDH may be implicated in inflammation-associated tissue injury and regeneration, which represents a common and clinically relevant situation in patients (in contrast to the rodent model of partial hepatectomy-associated liver regeneration). The goal of the current study was to assess the role of 15-PGDH and 15-keto-PGE_2_ in endotoxin-associated liver inflammation and tissue injury/regeneration. We developed liver specific 15-PGDH transgenic mice and the animals were subjected to LPS-induced liver inflammation/injury. To our surprise, we observed that the 15-PGDH transgenic mice were resistant to LPS-induced liver inflammation/injury. Mechanistically, our data showed that the 15-PGDH-derived 15-keto-PGE_2_ from hepatocytes activated PPAR-γ in Kupffer cells and thus inhibited their ability to produce inflammatory cytokines and that this paracrine mechanism led to attenuation of necro-inflammatory response in the liver. Our results provide evidence that 15-PGDH induction or 15-keto-PGE_2_ analogue may have therapeutic benefits in treatment of inflammation-associated liver injury.

## Results

### Liver specific expression of 15-PGDH protects mice from LPS-induced acute liver inflammation and tissue damage

**Fig 1:**
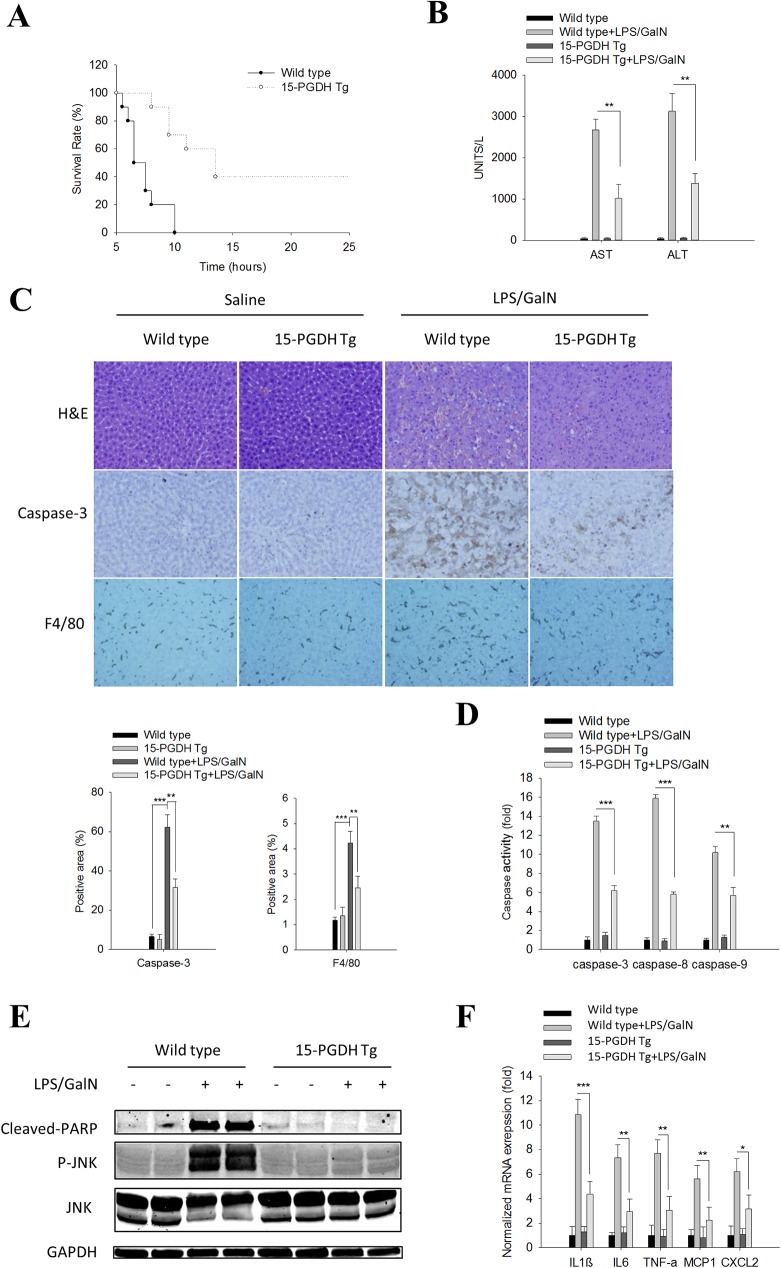
Liver specific 15-PGDH expression protects mice against LPS/GalN-induced acute liver inflammation and tissue damage. Wild type and 15-PGDH Tg mice were intraperitoneally injected with LPS (60ng/g body weight) plus GalN (800μg/g body weight). The mice were followed for survival or sacrificed at 5h after LPS/GalN injection to collect serum and liver tissue samples.(A) Survival curve of mice after LPS/GalN administration (n = 10). (B) Serum ALT and AST levels (n = 5). (C) Representative images of liver tissue sections (200×): H&E stain (upper panel), Caspase-3 IHC (mid panel), F4/80 IHC (lower panel). Quantified IHC results was shown below (n = 5). (D) Caspase-3, 8, and 9 activities in liver tissue homogenates (n = 3). The data were shown as fold changes compared to wild type mice. (E) The protein levels of apoptotic signaling molecules (Cleaved PARP, JNK, P-JNK) in liver tissue homogenates. GAPDH was used as loading control. (F) mRNA levels of pro-inflammatory cytokines (IL1β, IL6, TNF-α, MCP1 and CXCL2) in liver tissue homogenates (n = 3); the results are shown as fold changes compared to wild type mice. The quantitative data presented in this figure are expressed as means ± SE (*p<0.05, **p<0.01, ***p<0.001).

We developed transgenic mice with target overexpression of 15-PGDH in the liver by microinjection of a construct containing the 15-PGDH transgene under the control of albumin promoter/enhancer into fertilized mouse eggs of B6D2F1 mice at the single cell stage (Fig A in S1 File). To determine the effect of hepatic 15-PGDH on endotoxin-induced liver injury, the 15-PGDH Tg mice and their age/sex matched wild type mice were intraperitoneally injected with a single dose of LPS/GalN and the animals were monitored over 48h. We observed much prolonged survival of the 15-PGDHTgmice (ranging from 8h to >24h, with 50% survival at 12.5h) compared to the wild type control mice (ranging from 5.5h to 10h, with 50% survival at 6.5h) (Fig 1A). Based on the survival curves, we sacrificed additional mice 5h after LPS/GalN injection, and the blood and liver tissues were collected to evaluate parameters of liver injury. The15-PGDH transgenic mice had less prominent liver injury, as evidenced by lower serum ALT/AST levels (Fig 1B) and less hepatic necrosis/apoptosis (under H&E staining, caspase-3immunostaining, and caspase activity assays, and PARP cleavage) (**Fig 1C–1E**), compared to the wild type mice. We further observed that LPS/GalN treatment induced less hepatic inflammatory response in the 15-PGDH Tg mice, as reflected by the smaller population of F4/80 positive macrophages (Fig 1C**)** and the lower levels of pro-inflammatory cytokines (Fig 1F and Fig B in S1 File), compared to the matched wild type mice. Note that compared to the wild type controls, the 15-PGDH Tg mice showed marked reduction of serum AST (2727±264 IU/L in WT vs 975±237 IU/L in 15-PGDH Tg) and ALT (3138±435 IU/L in WT vs 1323±215 IU/L in 15-PGDH Tg).

Given that activation of JNK by TNF-α is a predominant mechanism for hepatocyte apoptosis in LPS/GalN-induced liver injury [13,14], we further measured the phosphorylation of JNK in the liver tissues. As shown in Fig 1E, overexpression of 15-PGDH in the liver completely prevented LPS/GalN-induced JNK phosphorylation. Taken together, these data provide novel evidence that the 15-PGDH Tg mice are resistant to LPS/GalN-induced acute liver inflammation/tissue damage.

### Hepatic 15-PGDH expression indirectly influenced Kupffer cell activation

**Fig 2:**
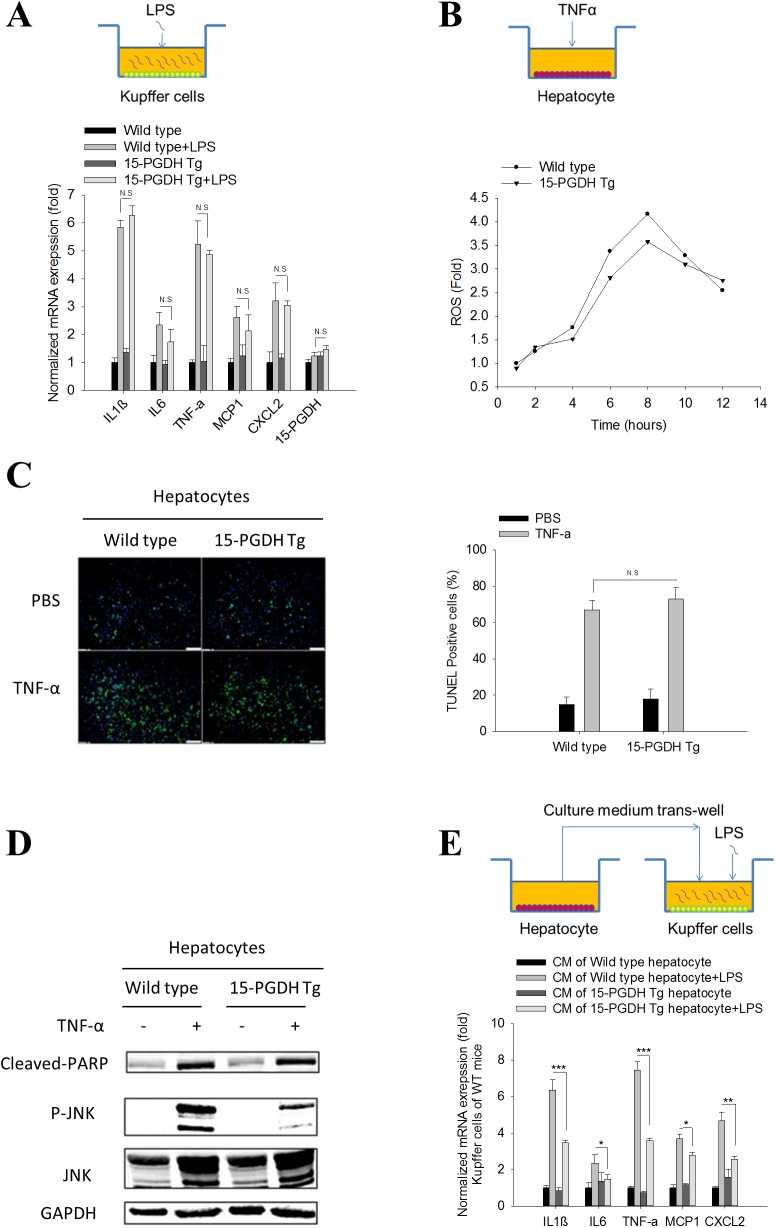
15-PGDH expression in hepatocytes regulates Kupffer cell cytokine production. Kupffer cells and hepatocytes were isolated and cultured separately. LPS (10ng/ml) was used to elicit Kupffer cell inflammatory response. TNF-α (25ng/ml) plus ActD (0.4μg/ml) was used to induce hepatocyte apoptosis. (A) mRNA level of pro-inflammatory cytokines (IL1β, IL6, TNF-α, MCP1 and CXCL2) and 15-PGDH in Kupffer cells after LPS treatment. The results are shown as fold changes compared to Kupffer cells isolated from wild type mice. (B) Accumulation of reactive oxygen species (ROS) in hepatocytes after TNF-α/ActD treatment. ROS was measured by dichlorofluorescin fluorescence assay and shown as fold change compared to untreated hepatocytes. (C) Hepatocyte apoptosis induced by TNF-α/ActD treatment. Apoptotic hepatocytes were stained by TUNEL assay. Representative images are showed in the left panel. Quantified results are showed in the right panel. (D) The protein levels of apoptotic signaling molecules (cleaved PARP, JNK, P-JNK) in hepatocytes after TNF-α/ActD treatment. GAPDH was used as loading control. (E) mRNA level of pro-inflammatory cytokines (IL1β, IL6, TNF-α, MCP1 and CXCL2) in WT Kupffer cells treated with hepatocyte CM followed by LPS stimulation. The results are presented as fold changes compared to Kupffer cells treated with CM of wild type hepatocytes. The quantitative data presented in this figure were obtained from three independent experiments and are expressed as means ± SE (*p<0.05, **p<0.01, ***p<0.001; N.S denotes no statistical significance).

The pathological processes of LPS/GalN-induced acute liver injury are well-described, in which LPS initially activates Kupffer cells to release a variety of pro-inflammatory cytokines, chemokines and ROS; these mediators can directly induce hepatocyte damage or cause liver injury by activating other inflammatory cells[15]. Among the inflammatory mediators, TNF-α is central in the development of liver injury, mainly through induction of hepatocyte apoptosis[13,14,16]. To delineate the contribution of Kupffer cells and hepatocytes, we isolated Kupffer cells and hepatocytes from the 15-PGDH Tg mice and matched wild type mice. We first treated cultured Kupffer cells with LPS and observed that LPS treatment induced the expression of several pro-inflammatory cytokines, including TNF-α, IL1β, IL6, MCP1 and CXCL2, in Kupffer cells from either wild type mice or 15-PGDH Tg mice (Fig 2A). Our data showed that the Kupffer cells isolated from wile type and 15-PGDH Tg mice responded similarly to LPS stimulation. We noted that 15-PGDH overexpression in hepatocytes did not significantly alter the expression of 15-PGDH in Kupffer cells (Fig 2A).

We next treated wild type or 15-PGDH-overexpressed hepatocytes with TNF-α (a predominant inflammatory cytokine released by Kupffer cells in response to LPS stimulation). Our data showed that the hepatocytes isolated from wild type and 15-PGDH Tg mice had a similar degree of JNK activation, ROS production and apoptosis in response to TNF-α treatment (Fig 2B–2D and Fig C in S1 File). Therefore, 15-PGDH expression in hepatocytes does not significantly alter their response to TNF-α.

To investigate the possible interaction between hepatocytes and Kupffer cells, we treated wild type Kupffer cells with the conditioned medium (CM) derived from wild type or 15-PGDH-Tg hepatocytes, and the Kupffer cells primed by the hepatocyte CM were then utilized to determine their response to LPS. We observed that the wild type Kupffer cells pre-incubated with the CM of 15-PGDH-Tg hepatocytes had less cytokine expression in response to LPS stimulation (compared to Kupffer cells pre-incubated with CM of wild type hepatocytes) (Fig 2E). These findings suggest that 15-PGDH in hepatocytes may regulate the production of soluble mediators which indirectly impact Kupffer cells response to LPS.

### 15-PGDH-derived 15-keto-PGE_2_ from hepatocytes inhibits Kupffer cell activation via PPAR-γ

**Fig 3:**
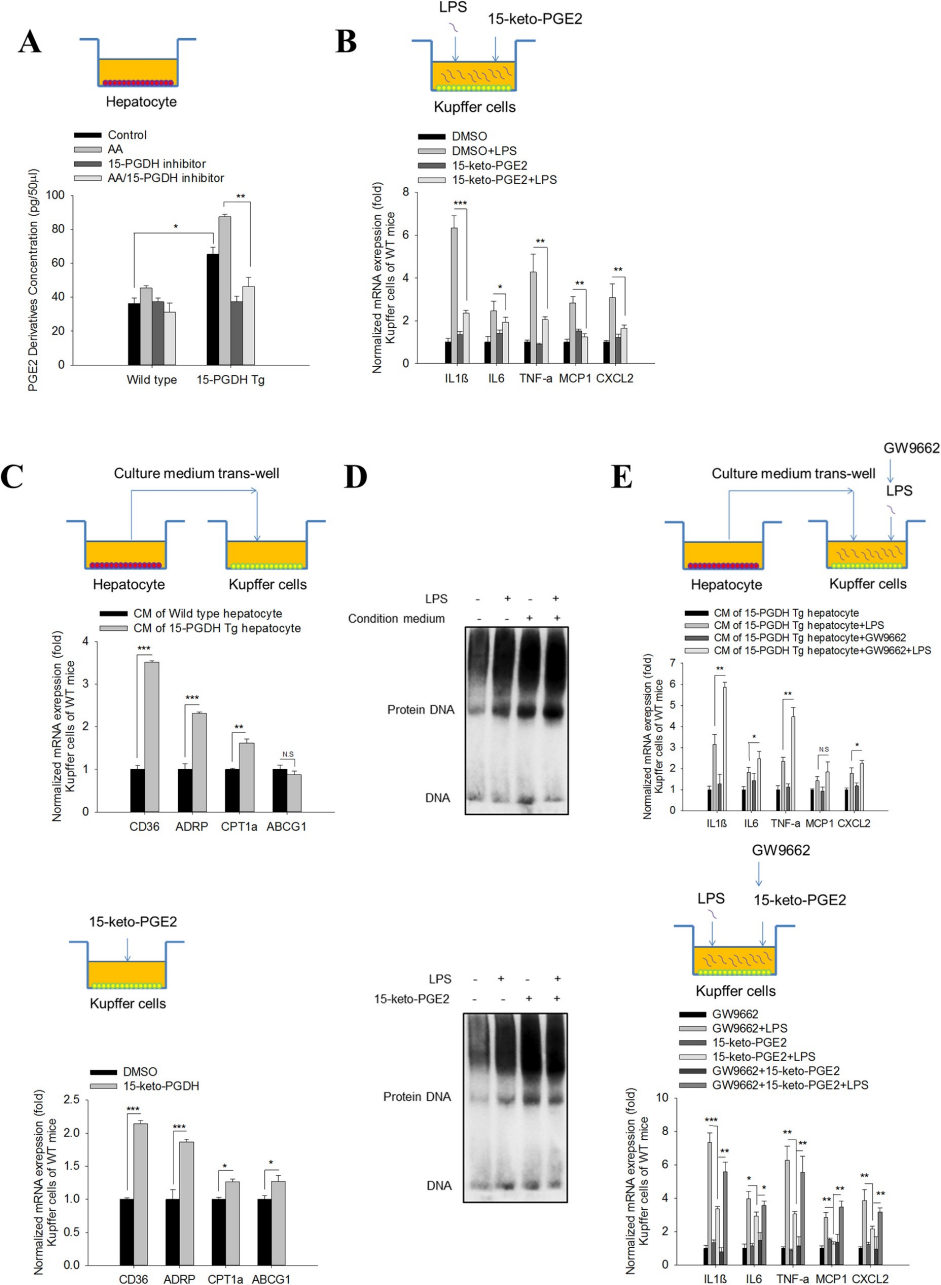
15-PGDH-derived 15-keto-PGE_2_ from hepatocytes inhibits Kupffer cell activation via PPAR-γ. (A) PGE_2_ metabolites concentration in CM of hepatocyte as measured by the Prostaglandin E Metabolite assay. We observed that 15-PGDH overexpression enhanced PGE_2_ metabolite production and this effect was more apparent in the presence of arachidonic acid substrate (1μM); this effect was blocked by treatment with the 15-PGDH inhibitor (1μM). (B) mRNA levels of pro-inflammatory cytokines (IL1β, IL6, TNF-α, MCP1 and CXCL2) in WT Kupffer cells treated with DMSO/15-keto-PGE_2_ (10μM) followed by LPS. The results are shown as fold changes compared to Kupffer cells treated with DMSO. (C) mRNA levels of PPAR-γ downstream genes (CD36, ADRP, CPT1α and ABCG1) in WT Kupffer cells treated with CM of hepatocytes (upper panel) or DMSO/15-keto-PGE_2_ (10μM) (lower panel). The data are shown as fold changes compared to Kupffer cells treated CM of wild type hepatocytes (upper panel) or Kupffer cells treated with DMSO (lower panel). (D) DNA binding ability of PPAR-γ from mouse macrophages (RAW264.7) treated with CM of hepatocytes (upper panel) or DMSO/15-keto-PGE_2_ (10μM) (lower panel), as determined by EMSA assay using PPRE dsDNA probe. (E) mRNA levels of pro-inflammatory cytokines (IL1β, IL6, TNF-α, MCP1 and CXCL2) in WT Kupffer cells treated with CM of hepatocytes (upper panel) or DMSO/15-keto-PGE_2_ (10μM) (lower panel) (with or without GW9662 [10μM] or LPS treatment). For the upper panel, the results are shown as fold changes compared to Kupffer cells treated with CM of 15-PGDH Tg hepatocytes. For the lower panel, the results are shown as fold changes compared to Kupffer cells treated with GW9662. The quantitative data in this figure were obtained from three independent experiments and are expressed as means ± SE (*p<0.05, **p<0.01, ***p<0.001; N.S—no statistical significance).

15-PGDH is well noted as the enzyme catalyzing conversion of pro-inflammatory PGE_2_ to its oxidized product,15-keto-PGE_2_, which is an endogenous PPAR-γ ligand [8,9,10,11]. Notably, PPAR-γ is a pivotal nuclear receptor that negatively regulates the production of pro-inflammatory cytokines in Kupffer cells [4,16,17]. Thus, we postulated that the 15-PGDH-derived 15-keto-PGE_2_ in hepatocytes may regulate Kupffer cell activation via PPAR-γ through a paracrine mechanism. In support of this, we observed significantly increased 15-keto-PGE_2_ production by15-PGDH-Tg hepatocytes compared to the wild type hepatocytes (especially when the cells were supplemented with the prostaglandin substrate, arachidonic acid (AA) (Fig 3A). The observation that the 15-PGDH inhibitor decreased 15-keto-PGE_2_ production in 15-PGDH Tg hepatocytes further support the role of 15-PGDH for 15-keto-PGE_2_ production. To further determine the impact of 15-keto-PGE_2_ on Kupffer cells, we pre-treated Kupffer cells with 15-keto-PGE_2_ prior to LPS stimulation. Our data showed that 15-keto-PGE_2_ treatment significantly inhibited LPS-induced cytokine expression in Kupffer cells (Fig 3B, Fig D in S1 File). We observed that 15-keto-PGE_2_ and CM of 15-PGDH-Tg hepatocytes exhibited comparable inhibitory effect on Kupffer cell activation. These findings strongly support hepatocyte-derived 15-keto-PGE_2_ for inhibition of Kupffer cell activation.

We sought to further determine the role of PPAR-γ in this process, and found that treatment of wild type Kupffer cells with15-keto-PGE_2_ or the CM of 15-PGDH Tg hepatocytes increased the expression of the PPAR-γ down-stream genes (CD36, ADRP, CPT1a, ABCG1) (Fig 3C). To determine whether 15-keto-PGE_2_ or 15-PGDH Tg hepatocyte CM might alter PPAR-γ protein binding to its DNA response element, we performed gel electrophoresis mobility shift assay (EMSA) in a mouse macrophage cell line (RAW264.7) using oligo-nucleotide corresponding to PPRE (PPAR response element). We observed that treatment of RAW264.7 cells with 15-keto-PGE_2_ or15-PGDH Tg hepatocyte CM enhanced PPAR-γ binding to PPRE (Fig 3D). We further utilized a PPAR response element (PPRE)-luciferase reporter system to assess the effect of 15-keto-PGE_2_ on PPAR-γ activation. Specifically, RAW264.7 cells were transfected with the PPRE-luciferase reporter vector followed by treatment with 15-keto-PGE_2_, vehicle control, or an endogenous PPAR-γ ligand, 15d-PGJ_2_. Our data showed that both 15-keto-PGE_2_ and 15d-PGJ_2_ enhanced the PPRE reporter activity (Fig E in S1 File). These findings support that 15-PGDH-derived 15-keto-PGE_2_ from hepatocytes can activate PPAR-γ in macrophages. The latter assertion is further supported by the observation that the PPAR-γ antagonist, GW9662, reversed the inhibition of Kupffer cell activation by 15-keto-PGE_2_ and by CM of 15-PGDH Tg hepatocytes (Fig 3E, Fig D in S1 File).

LPS stimulates Kupffer cell activation predominantly through the canonical TLR4-NFκB pathway [18]. To address whether 15-keto-PGE_2_ might interfere with NFκB activation, we pretreated RAW264.7 cells with 15-keto-PGE_2_ or DMSO vehicle control prior to LPS stimulation. Our data showed that 15-keto-PGE_2_ treatment did not significantly alter the expression of TLR4 or its adaptor protein MyD88 and did not influence LPS-induced NFκB phosphorylation (Fig F in S1 File). Thus, 15-keto-PGE_2_ does not appear to influence LPS-induced TLR4-NFκB signaling in our system. Together, our findings suggest that 15-PGDH-derived 15-keto-PGE_2_ from hepatocytes inhibits Kupffer cell activation by binding to PPAR-γ and that this mechanism may explain the resistance of 15-PGDH Tg mice to LPS/GalN induced liver injury.

### The PPAR-γ antagonist, GW9662, restored 15-PGDH Tg mice susceptibility to LPS-induced acute liver inflammation and tissue damage

**Fig 4:**
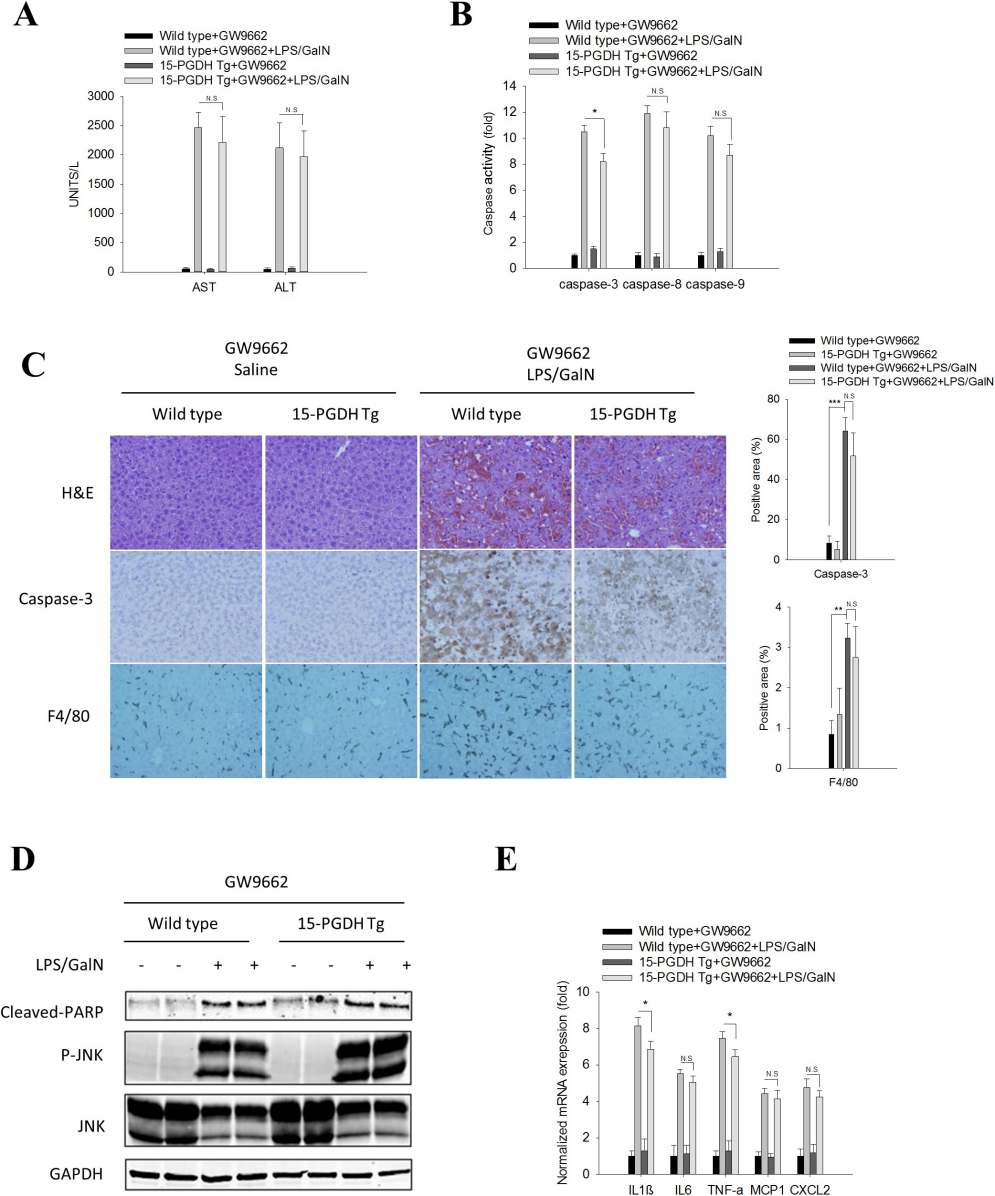
PPAR-γ antagonist, GW9662, restored the susceptibility of 15-PGDH Tg mice to LPS/GalN-induced acute liver injury. Wild type and 15-PGDH Tg mice were intraperitoneally injected with GW9662 (1μg/g body weight) 2 h before LPS/GalN administration. Mice were sacrificed 5 h after LPS/GalN injection to collect serum and liver tissue samples. (A) Serum ALT and AST levels (n = 3). (B) Caspase-3, 8, and 9 activities in liver tissue homogenates (n = 3). The data are shown as fold changes compared to wild type mice treated with GW9662. (C) Representative images of liver tissue sections (200×): H&E stain (upper panel), caspase-3 IHC (mid panel), F4/80 IHC (lower panel). Quantified results are showed at the right panels (n = 3). (D) The protein levels of apoptotic signaling molecules (cleaved PARP, JNK, P-JNK) in liver tissue homogenates. GAPDH was used as loading control. (E) mRNA levels of pro-inflammatory cytokines (IL1β, IL6, TNF-α, MCP1 and CXCL2) in liver tissue homogenates (n = 3). The results are shown as fold changes compared to wild type mice treated with GW9662. The quantitative data in this figure are expressed as means ± SE (*p<0.05, **p<0.01, ***p<0.001; N.S—no statistical significance).

To further verify the role of PPAR-γ, we administered the PPAR-γ antagonist, GW9662, to 15-PGDH Tg mice prior to LPS/GalN administration. We observed that GW9662 pretreatment reversed the resistance of 15-PGDH Tg mice to LPS/GalN-induced acute liver injury. As shown in Fig 4A and 4C, LPS/GalN-induced increase of serum ALT/AST and hepatocyte necrosis/apoptosis were comparable between the GW9662 pretreated 15-PGDH Tg mice and wild type mice. Likewise, the LPS/GalN-induced JNK phosphorylation, PARP cleavage and activation of Caspase3/8/9 were also comparable between the GW9662 pretreated 15-PGDH Tg mice and wild type mice (Fig 4B and 4D). Our data showed thatGW9662 treatment re-established the susceptibility of 15-PGDH Tg mice to LPS/GalN induced acute liver inflammation (as reflected by macrophage populations and cytokine levels) (Fig 4C and 4E). Collectively, these findings demonstrate that inhibition of PPAR-γ by its antagonist GW9662 can restore the susceptibility of the 15-PGDH Tg mice to LPS/GalN induced acute liver inflammation/injury.

## Discussion

15-PGDH is known to catalyze the oxidation of PGE_2_, a potent pro-inflammatory and pro-proliferative lipid mediator, to 15-keto-PGE_2_. While 15-keto-PGE_2_ had been long considered as an inactive metabolite of PGE_2_, recent studies have shown that 15-keto-PGE_2_ is biologically active by serving as an endogenous PPAR-γ ligand [8,9,10,11]. The current study provides novel evidence that 15-keto-PGE_2_ signal via PPAR-γ has an anti-inflammatory property in the setting of endotoxin-associated liver inflammation/injury. We show that after LPS/GalN injection, the 15-PGDH Tg mice exhibit much less necro-inflammatory response and less liver tissue damage compared to the wild type mice. This phenomenon is explained by the fact that 15-PGDH-derived 15-keto-PGE_2_ from hepatocytes is able to inhibit LPS-induced cytokine production in Kupffer cells, as depicted in the current study.

Our findings suggest that 15-PGDH-derived 15-keto-PGE_2_ from hepatocytes is able to attenuate endotoxin-induced liver inflammation/injury via activation of PPAR-γ in Kupffer cells. This assertion is consistent with the previous studies[4,19,20] documenting an anti-inflammatory effect of PPAR-γ in Kupffer cells (where PPAR-γ inhibits the production of inflammatory cytokines). In this context, it is worth mentioning that PPAR-γ has been well documented to inhibit the expression of pro-inflammatory genes in macrophages [21,22,23,24]. Our data presented in the current study are also consistent with a recent *in vivo* study [6] showing that mice with targeted deletion of PPAR-γ in Kupffer cells developed exacerbated response to CCl4-induced liver injury (characterized by higher necro-inflammatory activity, more prominent liver tissue injury and aggravated fibrogenic response). In our system, we show that 15-keto-PGE_2_ is able to activate PPAR-γ in mouse macrophages, as indicated by the fact that 15-keto-PGE_2_ enhances the DNA binding ability of PPAR-γ, increases the PPRE reporter activity and induces the expression of PPAR-γ down-stream genes. Further, our data indicate that the PPAR-γ antagonist, GW9662, is able to reverse 15-keto-PGE_2_-induced inhibition of cytokine production in Kupffer cells *in vitro* and can also restore the susceptibility of 15-PGDH Tg mice to LPS/GalN-induced liver injury *in vivo*. Taken together, these findings disclose a novel interaction between 15-PGDH/15-keto-PGE_2_ in hepatocytes and PPAR-γ signaling in Kupffer cells which coordinately regulate endotoxin-associated liver inflammation/injury.

In essence, the current study demonstrates that hepatic 15-PGDH protects against endotoxin-induced acute liver injury. Our findings point toward the possibility of 15-PGDH induction or 15-keto-PGE_2_ analogue as potential therapy for the treatment of inflammation-associated liver injury. In this context, it is worth mentioning that Zhang and colleagues [12] recently describe that 15-PGDH negatively regulates liver and colon tissue regeneration; the authors suggest that 15-PGDH inhibition may represent a therapeutic strategy to enhance tissue repair after injury under certain clinical contexts. In the current study we show that 15-PGDH in the liver actually attenuates endotoxin-associated liver inflammation and tissue injury. Our data suggest that induction, rather than inhibition, of 15-PGDH may have therapeutic value for the treatment of inflammation-associated liver injury. This viewpoint may have important clinical implication, given that inflammation-associated tissue injury and repair is pivotal in the pathogenesis of liver diseases associated with various underlying causes. Thus, whether 15-PGDH ought to be induced or inhibited for therapy is context-dependent and requires careful consideration of the underlying liver diseases.

## Materials and methods

### Ethical statement

All animal procedures were carried out in strict accordance with the National Institutes of Health Guidelines for the Care and Use of Laboratory Animals. The handling of the mice and all experimental procedures were specifically approved for this study by the Institutional Animal Care and Use Committee of Tulane University (IACUC's for the Tulane University Downtown Campus, Protocol #: 4159).

### Animal studies

Transgenic mice with expression of human 15-PGDH gene in hepatocytes were developed by pronuclear injection of 15-PGDH transgene construct into fertilized mouse eggs of B6D2F1 background at the single cell stage. Specifically, 15-PGDH transgene consisted of Hu-15-PGDH cDNA ligated to mouse albumin promoter/enhancer. The construct was microinjected into the pronuclei during the window of time the egg are visible within the protoplasm. The injected eggs were then transferred into the oviducts of pseudo pregnant foster mice. The pups born to the foster mothers with genomic integration of the injected DNA were identified by using tail DNA samples and the positively identified mice were selected as the transgenic founder. The Founder was then bred with the C57BL/6 wild type mice for more than five generations to produce incipient congenic 15-PGDH Tg mice (B6, ALB-hu-15-PGDH). The mice used in the study were F6 or subsequent generations of age-matched littermates.

For endotoxin-induced acute liver injury, mice (8–10 weeks old, female) were administered intraperitoneally with 60ng/g body weight lipopolysaccharides (LPS) (Sigma-Aldrich, St. Louis, MO) in combination with 800μg/g body weight of D-galactosamine (GalN)(Sigma-Aldrich, St. Louis, MO) (dissolved in0.9% sodium chloride solution). After LPS/GalN injection, the mice were followed for 48h for survival analysis or sacrificed at 5h to obtain blood and liver tissue samples.

For survival experiments, humane endpoints were applied to this study. The moribund states were used as humane endpoints. The following criteria have been used to identify moribund states: a) Inability to roll over from side to chest; b) Dyspnea, Labored breathing; c) Hypothermia. For liver sample collection, all mice were sacrificed by CO_2_ asphyxiation. The health of the animal was monitored every day. All efforts were made to minimize the distress and suffering of the animals.

### Isolation and culture of primary mouse liver cells

Hepatocytes were isolated by an adaptation of the calcium two-step collagenase perfusion technique as described previously[25]. Collagen I coated plates and dishes were purchased form BD Biosciences (San Jose, CA). 1×106, 3×106, or 2.5×104 hepatocytes were plated onto collagen-coated 6-well plates, 10-cm dishes, or 96-well plates, respectively. Hepatocytes were maintained in Williams’ Medium E medium (Invitrogen, Carlsbad, CA) supplemented with Hepatocyte Maintenance Supplement Pack (Invitrogen, Carlsbad, CA), 10% fetal calf serum (Sigma-Aldrich, St. Louis, MO), 2mM L-Glutamine (Invitrogen, Carlsbad, CA) and Antibiotic-Antimycotic (Invitrogen, Carlsbad, CA). Cultured hepatocytes were treated with TNF-α (R&D systems, Minneapolis, MN) (25 ng/ml) plus Actinomycin-D (ActD) (Thermo Fisher, Waltham, MA) (0.4 μg/ml) for 8h for induction of apoptosis.

For Kupffer cell isolation, collagenase-perfused liver tissues were further digested in RPMI-1640 (life technology, Grand Island, NY) containing 0.1% type IV collagenase (Roche, Indianapolis, IN) for 30min at 37°C. The liver homogenate was filtered and centrifuged at 50×g for 5min. The top aqueous phase was reserved and centrifuged at 1400×g for 10min; the cell sediment mainly contained Kupffer cells. The cells were suspended and maintained in 6-well plate at a density 1–3×106 /well in DMEM (life technology, Grand Island, NY) supplemented with 10% heat-inactivated fetal bovine serum (Sigma, St. Louis, MO).

For treatment with hepatocyte conditioned medium (CM), the CM was collected from hepatocyte cultures and filtered through a 0.22-μm sterile filter; the CM was then diluted 1:1 (vol/vol) with Dulbecco's modified Eagle medium containing 5% FBS before addition to cultured Kupffer cells.

Cultured Kupffer cells were pretreated with either CM of hepatocytes or DMSO/15-keto-PGE_2_ (Cayman,Ann Arbor, MI) for 6h before LPS stimulation. LPS (10ng/ml) were used to activate Kupffer cell inflammatory response. The mRNA levels of inflammatory cytokines were measured by qRT-PCR 6h after LPS treatment.

### ALT/AST analysis

Blood samples were centrifuged at 3000rpm for 15min to obtain serum. Serum alanine aminotransferase (ALT) and aspartate aminotransferase (AST) levels were measured with an automatic analyzer at the Department of Clinical Chemistry, Tulane University Hospital.

### Cytokine analysis

The liver tissue was homogenized in PBS containing protease inhibitors (Roche, Indianapolis, IN). The obtained supernatants were stored at −80°C and analyzed for 26 cytokine levels at Stanford Human Immune Monitoring Center by the Luminex mouse cytokine bead immunoassay kit (Birsource, Camarillo, CA) according to the manufacture's instructions.

### Immunohistochemical (IHC) procedure

Liver samples were fixed in 10% buffered formalin and embedded in paraffin. Sections (4μm) were deparaffinized and processed for hematoxylin and eosin (H&E) staining, Trichrome staining and immunohistochemistry. Antibodies were diluted in 1×PBS containing 4% horse serum, 0.2% Triton-X100 and 0.4mg/ml merthiolate. 15-PGDH antibody (1:500, Novus Biologicals, Littleton, CO) was used to detect 15-PGDH expression. Cleaved caspase-3 antibody (1:200, Biocare Medical, Pike Lane Concord, CA) was used to detect apoptotic cells. F4/80 antibody (1:200, abcam, Cambridge, MA) was used to detect activated macrophages. Horseradish peroxidase–conjugated goat anti-rabbit IgG was used as the secondary antibody. Signals were visualized using 0.2mg/mL diaminobenzidine, 0.01% hydrogen peroxide in 0.1 M phosphate buffer.

### Caspases activities analysis

Liver protein extracts were prepared as previously described[26]. Caspase-3/7, caspase-8 and caspase-9 activities were measured with Caspase-Glo Assay kit (Promega Corporation, Madison, WI). The caspase activities were expressed as fold changes over the control (corresponding wild type mice).

### Prostaglandin E metabolite assay

Isolated hepatocytes were cultured in serum-free medium with/without arachidonic acid (AA) (Cayman,Ann Arbor, MI) (1μM) and/or 15-PGDH inhibitor (EMD Millipore, Billerica, MA) (1μM) for 6h. Before collecting culture medium, cells were stimulated with Calcium ionophore A23187 (Sigma,St. Louis, MO) (10μM) for 10min. Prostaglandin E Metabolite concentration in culture medium was analyzed according to the instruction of Prostaglandin E Metabolite EIA Kit (Cayman, Ann Arbor, Michigan).

### Real-time PCR

Total RNA was extracted from either liver tissue samples or isolated Kupffer cells according to TRIzol® Reagent method (life technology, Grand Island, NY). mRNA levels were quantified by using RT^2^ SYBR® Green qPCR kit (QIAGEN, Germantown, MD); GAPDH is used as internal control. Primers used are listed in Table A in S1 File.

### Western blot analysis

Liver tissue samples or isolated liver cells were homogenized and lysed by NP-40 lysis buffer. All lysis buffers were prepared with the protease inhibitor cocktail and phosphatase inhibitor cocktail (Roche, Indianapolis, IN). Cellular proteins were separated by SDS-PAGE and transferred onto nitrocellulose membranes (Bio-Rad, Hercules, CA). The membranes were blocked by PBS-T (0.5% Tween 20 in PBS) containing 5% nonfat milk for 1h at room temperature, and then incubated with individual primary antibodies in PBS-T containing 5% nonfat milk for 2-5h at room temperature with the dilutions specified by the manufacturers. Following three washes with PBS-T, the membranes were incubated with IRDye 680LT/IRDye 800CW secondary antibodies (LI-COR Biosciences, Lincoln, NE) in PBS-T for 1h at room temperature. The membranes were then washed with PBS-T and the protein bands were visualized by using the ODYSSEY infrared imaging system (LI-COR Biosciences, Lincoln, NE).

### TUNEL assay

Hepatocytes were fixed in 4% formaldehyde for 25min at 4°C. TUNEL staining of fixed cells was performed using DeadEnd™ Fluorometric TUNEL System (Promega, Madison, WI). The apoptotic index was calculated as the percentage of positively stained cells.

### ROS assay

Hepatocytes were seeded at 2.5×104 cells/well on 96 well plates. ROS Assay of adherent cells was performed using DCFDA Cellular ROS Detection Assay Kit (Abcam, Cambridge, MA). Signal was measured at 485 nm/535 nm by FLUOstar Omega (BMG labtech, Cary, NC). The data are expressed as percentage of fold change.

### EMSA

Nuclear protein was prepared by nuclear extraction kit (EMD Millipore, Billerica, MA). EMSA was processed according to the **protocol** of The Gelshift Chemiluminescent EMSA Assay Kit (Active Motif, Carlsbad, CA). The oligonucleotide used as PPAR-γ probe is listed in Table A in S1 File.

### Luciferase reporter assay

2×105 RAW264.7 cells were plated in 6-well plate and cultured overnight. Cells were co-transfected with PPAR-γ response element (PPRE)-luciferase reporter (Addgene) and pRL-TK (Renilla luciferase expression vector, act as an internal control) (Addgene). After 16 hours of transfection, the cells were treated with vehicle control (DMSO), 15d-PGJ_2_ (3μM) or 15-keto-PGE_2_ (10μM) for 24 hours. Cells were harvested and luciferase activities of the cell extracts were measured using the dual luciferase assay system (Promega). The PPRE luciferase activity (firefly) was normalized to Renilla luciferase expression.

### Statistical analysis

Data are presented as mean ± standard error. Differences between two groups were determined by a two-tailed Student’s t test. Kaplan-Meier survival analysis was used for mortality analysis. A value of p<0.05 was considered to be statistically significant.

## Supporting information

S1 File Supplementary figures and table. (DOCX)
