# Supplementary material for: 15-hydroxyprostaglandin dehydrogenase (15-PGDH) prevents lipopolysaccharide (LPS)-induced acute liver injury
Source: PLoS One. 2017 Apr 19;12(4):e0176106. doi: 10.1371/journal.pone.0176106 (PMC5397067; doi:10.1371/journal.pone.0176106)

**Supplementary figure legends**

**Fig A. Development of liver specific 15-PGDH Tg mice.** (A) Schematic presentation of the strategy to develop 15-PGDH transgenic mice as described in the Methods. (B) The expression of 15-PGDH in liver tissues from wild type and Tg mice. (Left panel) IHC stain for 15-PGDH in liver tissue sections. (Right panel) 15-PGDH protein level in liver tissue homogenates (GAPDH as loading control).

**Fig B. Cytokines levels in liver tissues.** Wild type and 15-PGDH Tg mice were intraperitoneally injected with LPS (60ng/g body weight) plus GalN (800µg/g body weight). The liver tissue samples were collected 5h after LPS/GalN injection (n=3). The levels of 26 cytokines in the liver tissue homogenates were analyzed as described in the Methods.

**Fig C. The effect of 15-keto-PGE_2_ and GW9662 on TNF-α induced hepatocyte apoptosis.** (A) The protein levels of apoptotic signaling molecules (cleaved PARP, JNK, P-JNK) in hepatocytes with indicated treatments (WT hepatocytes were pretreated with 10µM 15-keto-PGE_2_ [left panel]; 15-PGDH Tg hepatocytes were pretreated with 10µM GW9662 [right panel]; the cells were then treated with TNF-α (25 ng/ml) plus ActD (0.4 µg/ml)). GAPDH was used as loading control. (B) ROS accumulation in hepatocytes after TNF-α/ActD treatment. ROS was measured by dichlorofluorescin fluorescence assay and expressed as fold change compared to hepatocytes treated with DMSO. (C) Hepatocyte apoptosis induced by TNF-α/ActD treatment. Apoptotic hepatocytes were stained by TUNEL assay. Representative images were showed in upper panel. Quantified results were showed in lower panel. The quantitative data were obtained from three independent experiments and the results are expressed as means ± SE (**p<0.01; N.S - no statistical significance).

**Fig D. 15-PGDH-derived 15-keto-PGE_2_ from hepatocytes inhibits Kupffer cell inflammatory response via PPAR-γ.** (A) Kupffer cells were isolated from the 15-PGDH Tg mice. The isolated Kupffer cells were treated with the indicated CM of hepatocytes followed by LPS stimulation. The levels of pro-inflammatory cytokine mRNAs in treated Kupffer cells were measured by qRT-PCR and the results are shown as fold changes (compared to Kupffer cells treated with CM of wild type hepatocytes). (B) Kupffer cells were isolated from the 15-PGDH Tg mice, and the isolated Kupffer cells were treated with 15-keto-PGE_2_ (10µM) or vehicle DMSO followed by LPS stimulation. The levels of pro-inflammatory cytokine mRNAs in treated Kupffer cells were measured by qRT-PCR and the results are presented as fold changes (compared to Kupffer cells treated with DMSO). (C) Kupffer cells were isolated from the 15-PGDH Tg mice, and the isolated Kupffer cells were treated with the indicated CM of hepatocytes (left panel) or DMSO/15-keto-PGE_2_ (10µM) (right panel) with or without GW9662 (10µM) or LPS. The levels of pro-inflammatory cytokine mRNAs in treated Kupffer cells were measured by qRT-PCR. For the left panel, the results are shown as fold changes compared to Kupffer cells treated with CM of 15-PGDHTg hepatocytes. For the right panel, the results are shown as fold changes compared to Kupffer cells treated with GW9662. *p<0.05, **p<0.01, ***p<0.001. N.S denotes no statistical significance.

**Fig E. 15-keto-PGE_2_ increases PPRE-luciferase activity.** RAW264.7 cells transfected with the PPRE-luciferase reporter plasmid and the thymidine kinase promoter-Renilla luciferase reporter plasmid (pRL-TK) were treated with DMSO, 15d-PGJ_2_ (3µM), or 15-keto-PGE_2_ (10µM) for 24 hours. Dual luciferase reporter activities were then determined. The PPRE luciferase reporter activity (firefly) was normalized to the Renilla luciferase activity (n=3, **p<0.01 compared to DMSO).

**Fig F. Effect of 15-keto-PGE_2_ on TLR4-NFκB signaling.** RAW264.7 cells were treated vehicle control (DMSO) or 15-keto-PGE_2_ (10µM) followed by LPS 100ng/mL for 30min, 1h and 4h. The levels of TLR4, MyD88 and p-NF-κB(Ser536) were analyzed by Western blotting. GAPDH served as an internal control. Data are representative of three independent experiments.


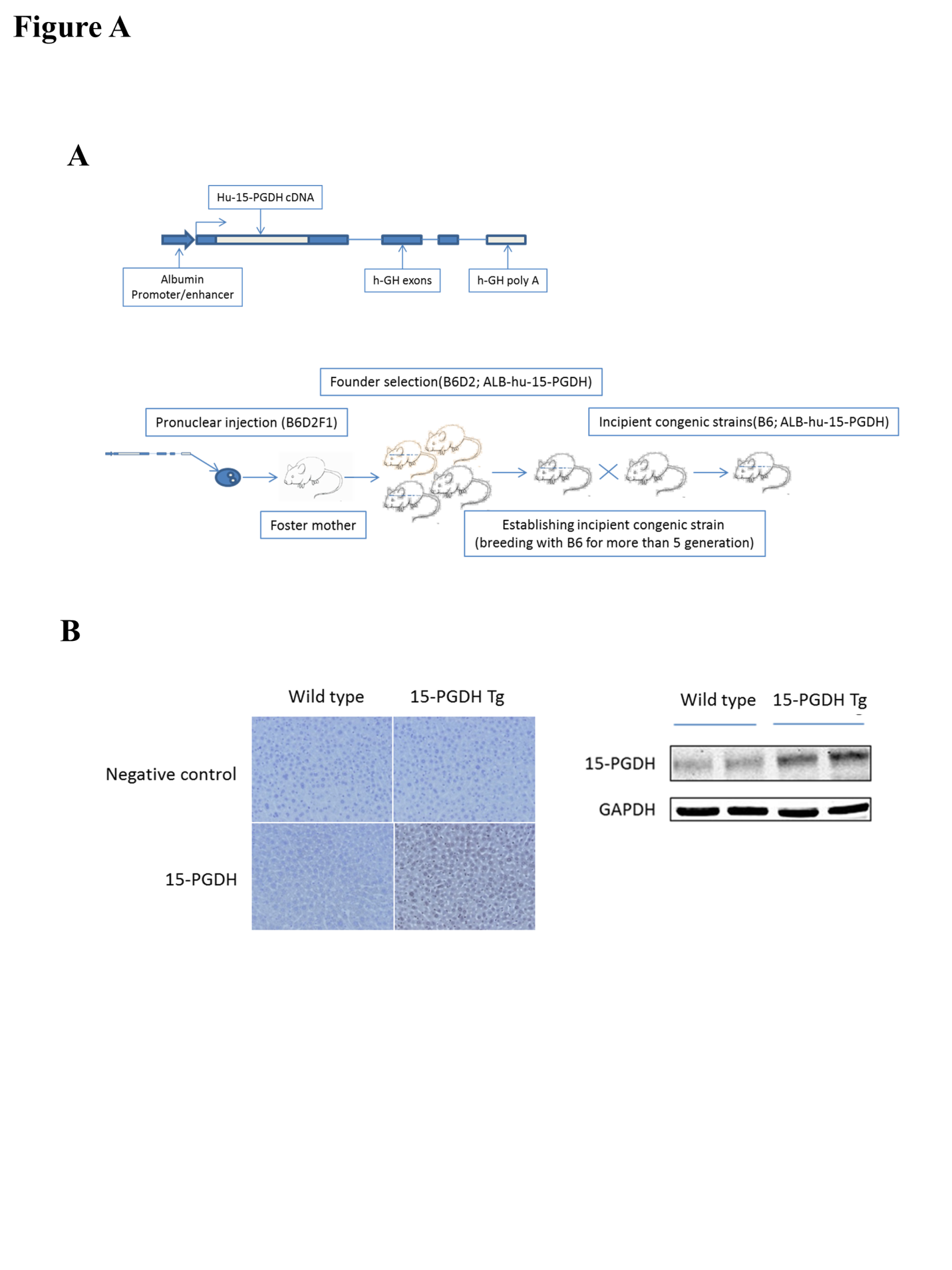

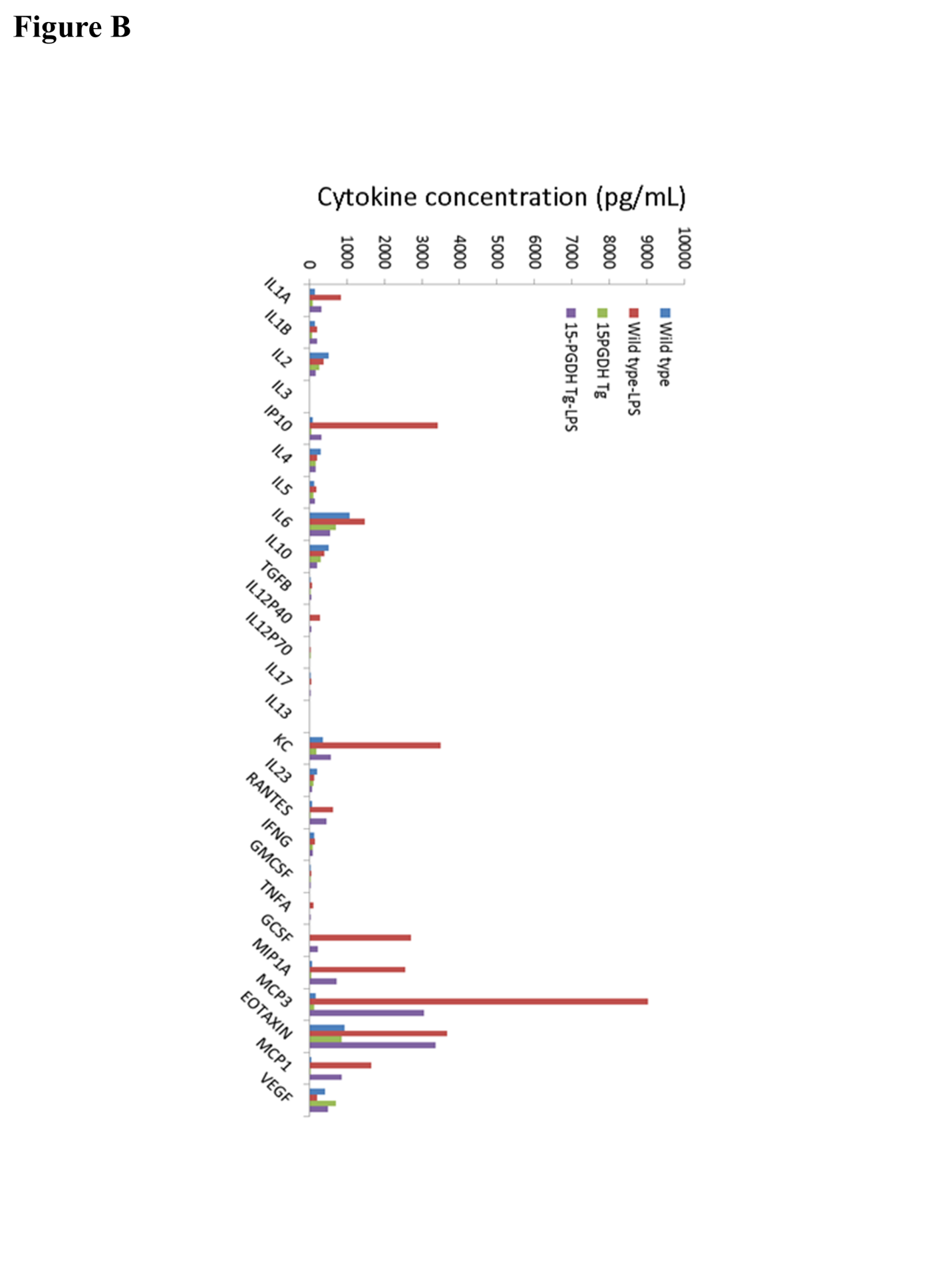

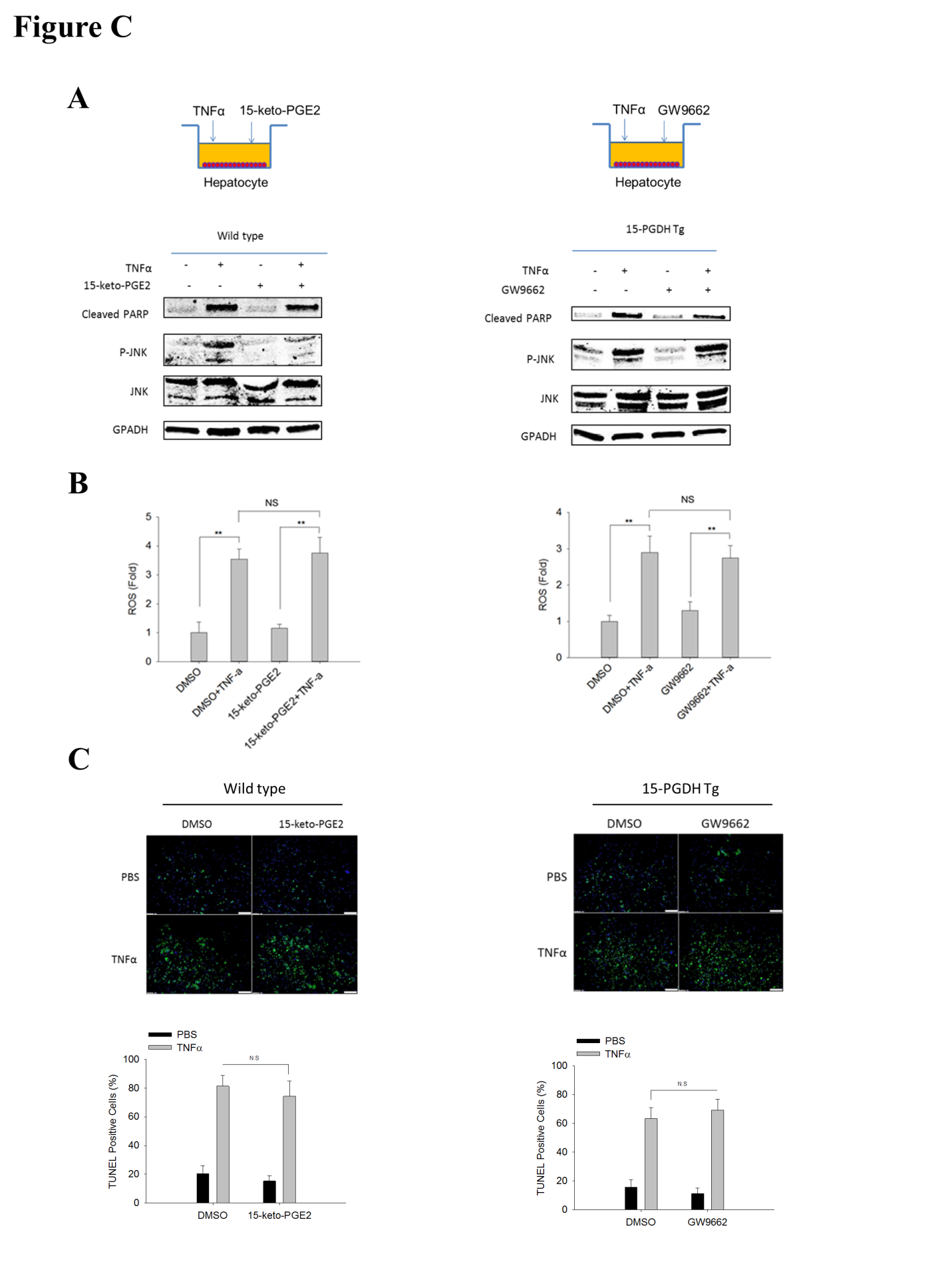

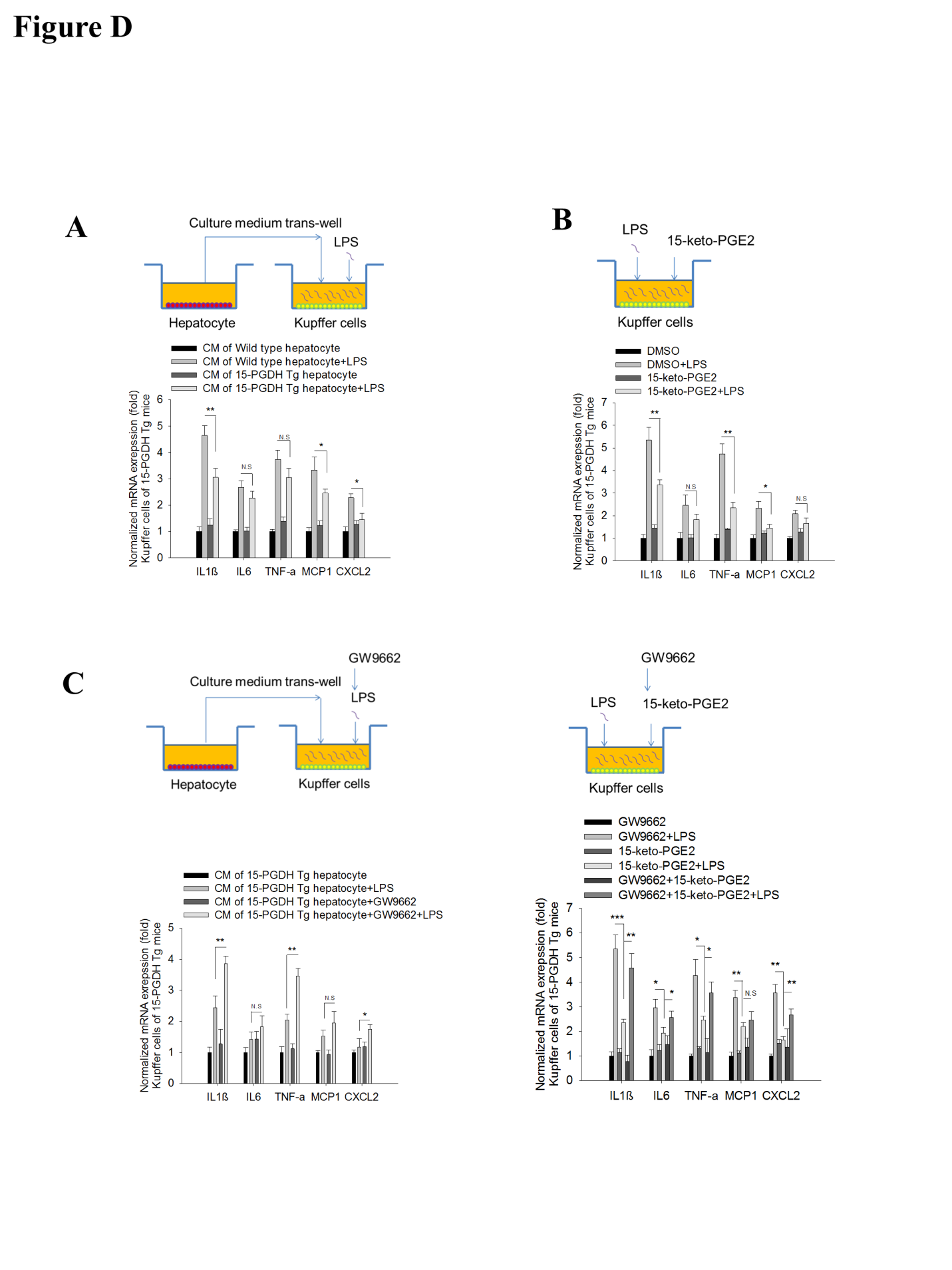

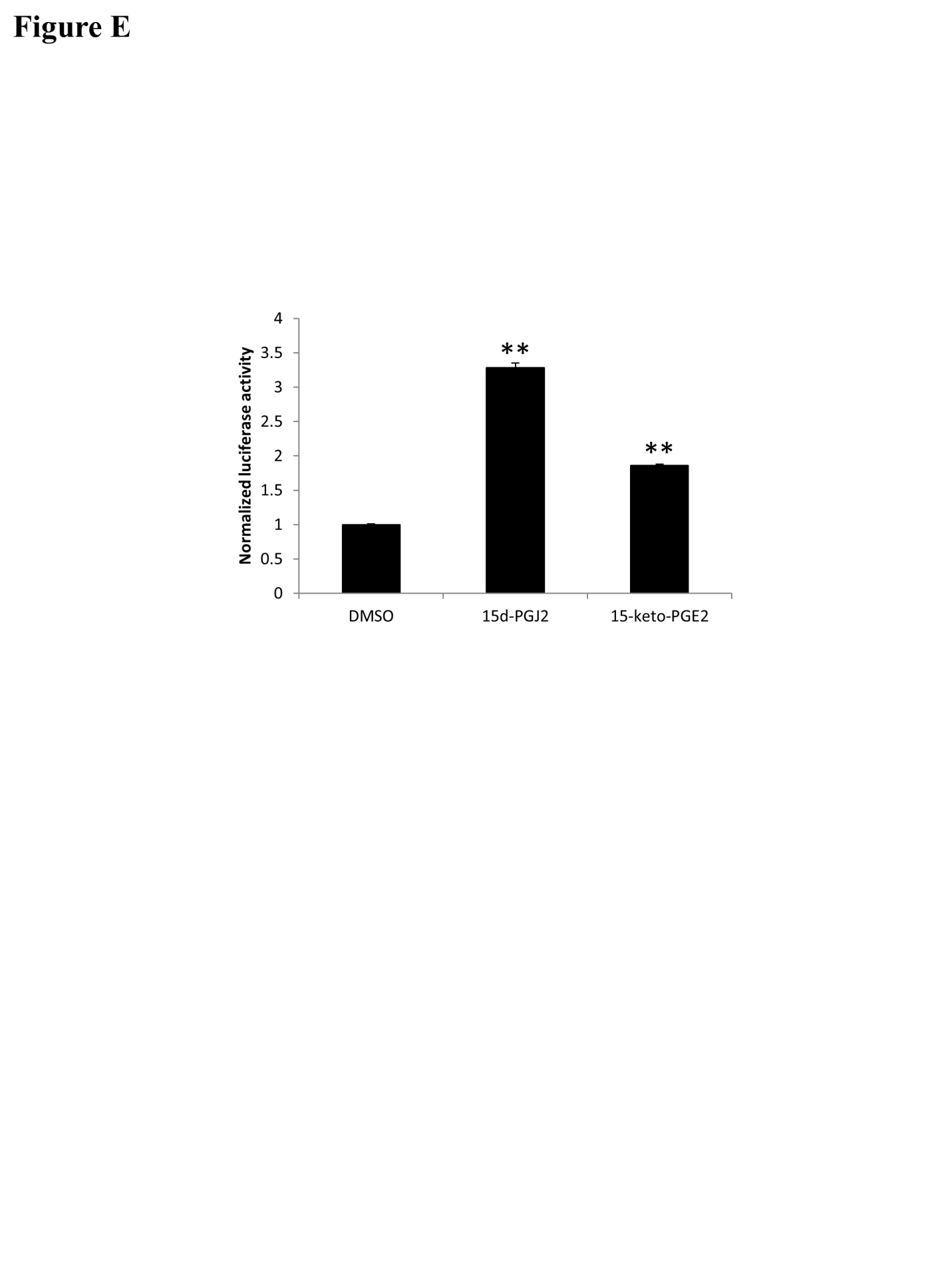

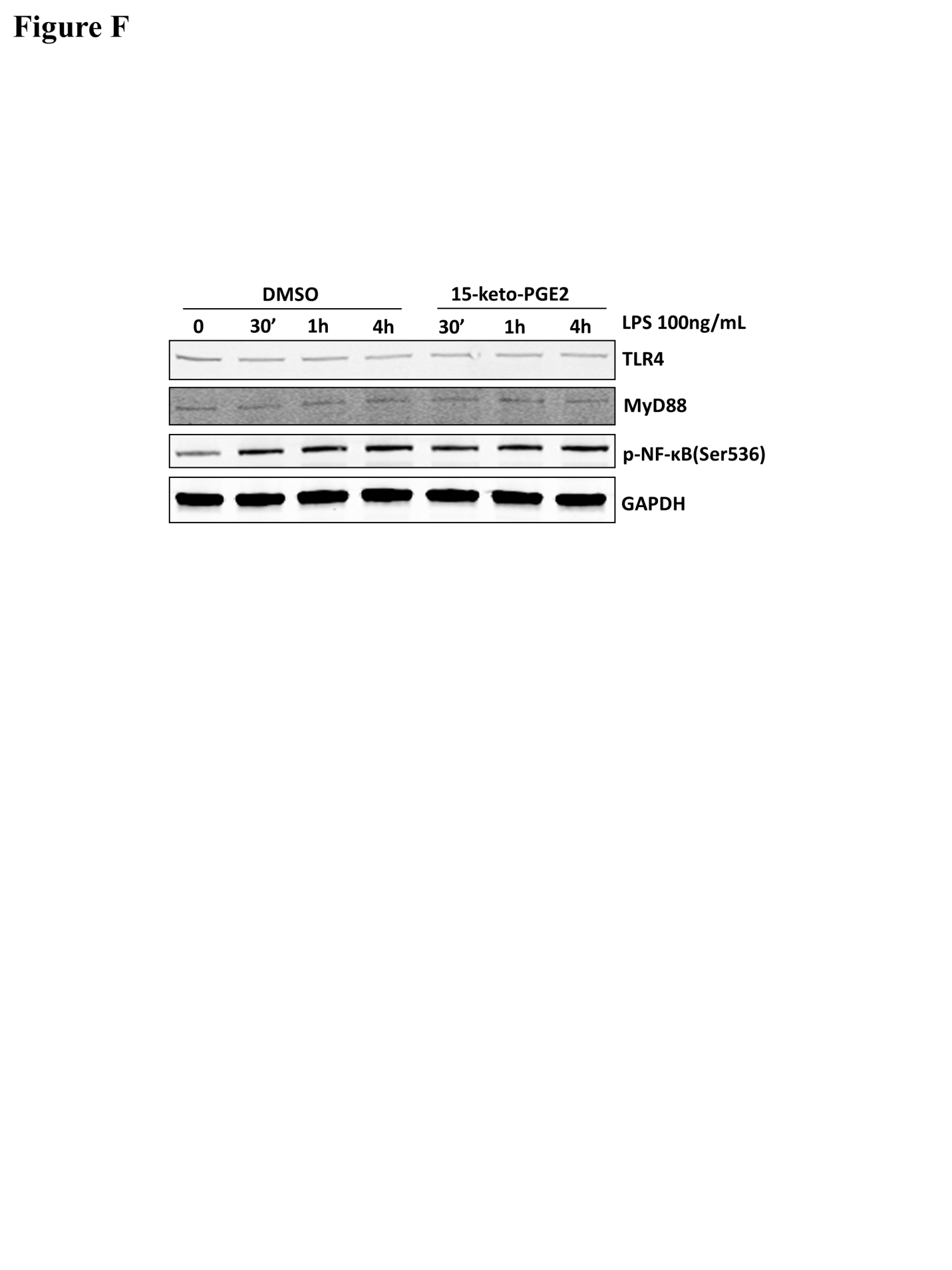

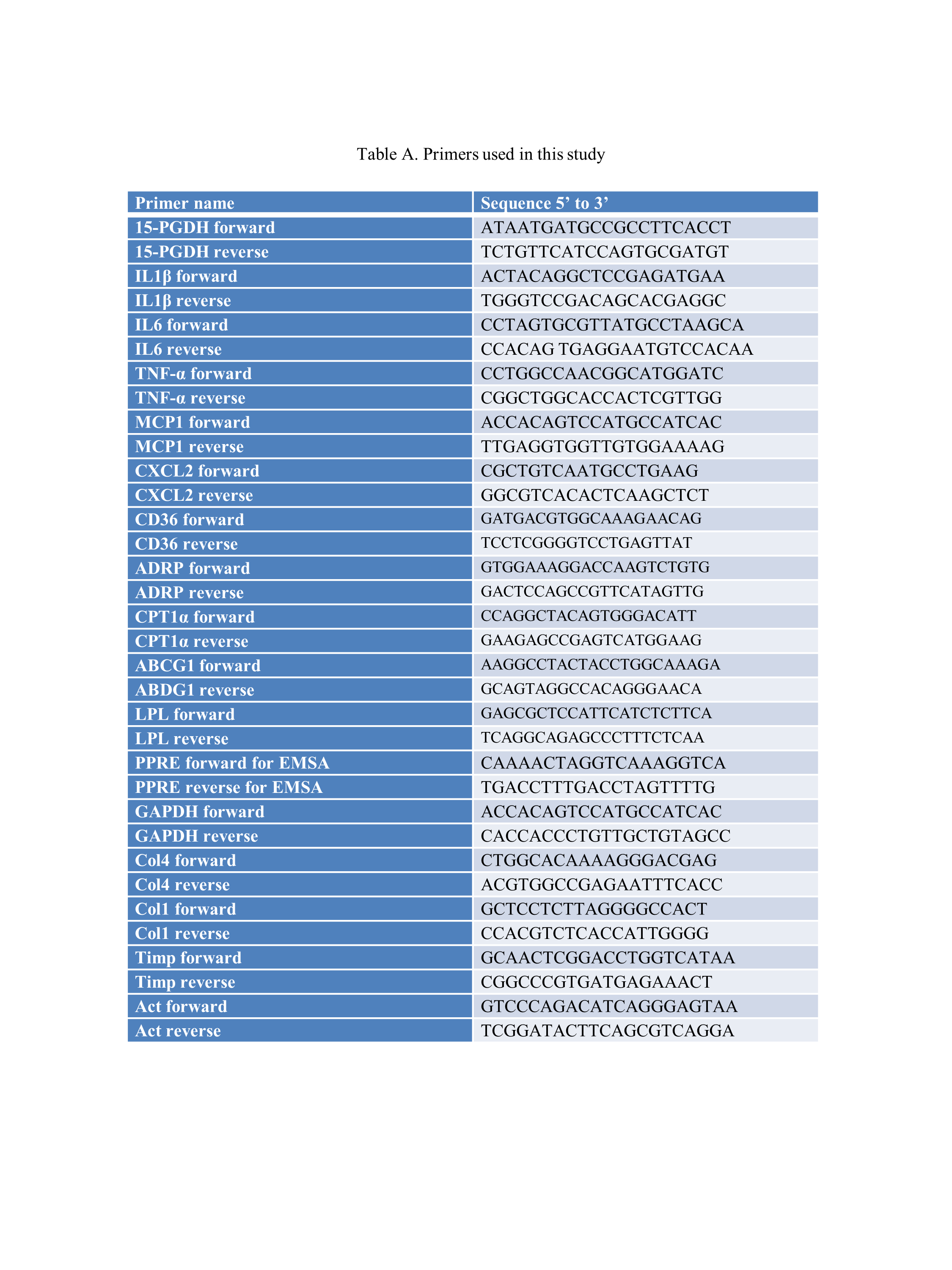

Supplement: S1 File — (DOCX) [file pone.0176106.s001.docx]
